# Progress, Liberty, Unity

**Published:** 1903-02

**Authors:** Henry Reed Hopkins

**Affiliations:** Buffalo, N. Y., 433 Franklin Street.


### BUFFALO MEDICAL JOURNAL.

VOL. XLII.—LVIII. FEBRUARY, 1903. No. 7.

### ORIGINAL COMMUNICATIONS.

### Progress, Liberty, Unity.

**Anniversary Address of the President at the Ninety-seventh Annual Meeting of the Medical Society of the State of New York.**[1]

By HENRY REED HOPKINS, M. D., Buffalo, N. Y.

THE submission of a free people to the executive authority of government is no more than a compliance with laws which they themselves have enacted.

The first requisite of liberty, as we and our forefathers have known it, is the willingness to abide by the law.

Fear nothing but to do wrong, dread nothing, but to be recreant to public duty.

A sympathetic study of the history of our society, a history which began nearly a hundred years ago; an intimate personal knowledge of its affairs for almost half of that time; a review of the general progress of medical discovery during the same period; a careful study of its medical legislation and litigations; some acquaintance with the coincident developments in political, economical, and sociological science, and meditation upon the contemporaneous movements of certain great medical bodies toward organisation or reorganisation on broad, catholic, legal principles, have convinced me that the medical mind is prepared as it never before has been for a judicious consideration of the important truths included in our title.

The facts I ask you to consider fall naturally into three groups,—the political, the practical, and the scientific.

#### POLITICAL.

I know of no duty, next to the worship and obedience which we owe to our Father who art in Heaven, that is more impera-

1. At Albany, N. Y., Wednesday, January 28, 1903.

tive in its requisitions and more delightful in the performance, than that which the municipal law requires from its various professors. To be shielded and led by the divine nature and immortal reason of law is one of the most sacred rights of man.

It seems to have been assumed by the state from the beginning, and the assumption has been consistently sustained by the courts, that as the state recognises religion, but no particular sect, so it recognises medicine, but no special school of practice. The state does not claim to be an expert in the fine distinctions of either religion or medicine, but freely concedes to the masters in both that priceless gift, the most precious in the power of any government to bestow, liberty—namely, liberty in worship and in science. Our constitution provides that the worship of Almighty God shall be free in thought, in heart, and in form; that in medicine belief and practice shall be free; its investigation unhampered, its conclusion uncontrolled by human authority.

Only by grasping the full significance of these two facts: (1) that the medical profession is a unit; (2) that it is free and its members independent, and holding them in equipoise without slighting or minimising either, may we prepare our minds for the right reception of the truths of this question, in sound discretion and for the furtherance of justice. So, and only so, can we prove that we have made good use of our liberty; so, and only so, can we demonstrate our fitness for American citizenship.

Our state constitution reads: The enacting clause of all bills shall be: The people of the State of New York, represented in Senate and Assembly, do enact as follows. We will do well to remember that the sanction and authority of law have lost nothing in force or in continuing effect by the transfer from the former style of We, by the Grace of God, King, Emperor, Most High Sovereign, Ruler and Potentate, to We, the people of the State of New York, grateful to Almighty God for our freedom, in order to secure its blessings do establish this constitution.

In the olden time obedience to law and reverence for it sprang from the conviction that the King's authority, the source of law, could do no wrong.

Shall not we who have enjoyed for more than a century the blessings of liberty repeat with equal fervor, with increased loyalty, with added appreciation, the words of Webster, Justice, sir, is the great interest of man on earth. It is the ligament which holds civilised beings and civilised nations together. Wherever her temple stands, and so long as it is duly honored, there is a foundation for social security, general happiness and the improvement and progress of our race; and the still more expressive apostrophe of Everett: Universal law—in action alternately executive, legislative, and judicial; in form successively constitution, statute, and decree—mingles into one harmonious, protecting, strengthening, vitalising sublime system; brightest image on earth of that ineffable Sovereign Energy, which, with mingled power, wisdom and love upholds and governs the universe. Again, it is only necessary to recall that the sovereign will of We, the people of the Empire State, has enacted that the medical profession shall have unity and liberty, and as we value liberty—that priceless blessing—let us recall that liberty is our birthright under the constitution, which we may magnify, honor and defend by obedience; and that effectiveness in progress, security in liberty, demand unity.

And further, we must not forget that responsibility is always measured by opportunity, that the strength and perpetuity of constitutional government are established and promoted in proportion as the whole people show sympathetic obedience unto the statutes and unto the judgments. We of the medical profession know more than the average citizen; knowledge increases responsibility. How may we better teach the ignorant artisan —half savage and part anarchist — self-control, moderation, respect and obedience to law, than by our own example of obedience, and of obedience even against inclination.

Moreover, I am sure that now, as in times past, misunderstanding plays a large part in the opposition of our profession to the efforts of the state in the direction of medical unity. We have feared that the state desired and sought to produce by legal enactment uniformity of medical practice. That is an impossibility. Men are individuals and individuals always have and always will differ. A clear perception that medical unity is not uniformity of practice, and that a proclamation of uniformity would be a denial of freedom and therefore impossible, should prevent further misunderstanding in this direction and enable us to appreciate rightly, legal medical unity, its origin, nature, and potentiality, to the end that we may conduct ourselves in harmony with its principles.

Unity in medicine is absolutely essential to efficiency and progress in that most important branch of sanitary science, state medicine. Consider for a moment some of the characteristics of state medicine. It is the supreme function of our profession. Success here means honor and confidence both at home and abroad and wins the sympathetic ear of the most liberal progressive and competent men. Nor should we forget that its work is never ended. Its problems increase in difficulty and complexity with the age of the government, the density of population, and the wealth of the people.

Sanitary codes,—the most intelligent and the most humane of legal enactments,—are the joint product of the will of the people and the wisdom of their physicians. Such codes and the manner of their execution are the most accurate exponents of the real civilisation of their times; their conception, enactment, and execution demand good understanding and sympathy between the parties.

Sanitary organisation should keep pace with the progress of preventive medicine, and so far as the conditions of the people permit, it should be the most complete, the most intelligent and the ablest of all in this age of organisations.

What could be more appropriate than that the Empire State by a united medical profession, in cordial sympathy with the law and with the source of law, should show the way to a sanitary organisation commensurate in dignity and completeness of detail with her exalted position and noble purposes, and evolve sanitary codes so perfect that the humblest child in the state might receive the fullest benefit of the wisest, most modern, and most progressive, preventive medicine.

I should be false to this occasion and its high responsibility if I failed to remind you that the sanitary organisations of our state, as well as those of her sister commonwealths, are distinctly inferior to those of contemporaneous Europe in quality of membership, in official rank, in public estimation and consequently in effectiveness.

The wife of the humblest moujik is attended in childbirth by a midwife, whose license runs in the name of We, Nicolos Alexandrovitch, Emperor and Autocrat of all the Russias, Tsar of Moscow, Lord of Pskow, Grand Duke of Smolensk. The state board of health of Germany is under the presidency of the Chancellor of the German Empire. Is it strange that the great cities of that empire have less than one-third of the typhoid fever that annually ravages the cities of this state?

The literature of state medicine contains no history more instructive or more inspiring than the account of the sanitary measures by which Hebrew slaves escaping from the brick-yards of Egypt were guarded during the whole of that marvelous march in the wilderness of Zin,—march not of a week, the time that seems to be the limit of strength and resources for our regiments,—but of forty years, the time required for the wisest sanitation to transform a mob of slaves into a nation of warriors, statesmen, architects, historians, poets, capable of establishing the civilisation of Palestine, of building the temple of Solomon, and of producing a monument more enduring than marble or brass, the literature of Isaiah and of David. But the head of their board of health was Moses, the man who talked with God face to face.

Our minds should be stimulated, our convictions strengthened, our energies aroused by the contemplation of the fact, that the glorious accomplishments of state medicine in the past have been achieved without aid from the science of hygiene; our fathers had only faith and hope,—we have also demonstrated knowledge. The introduction of pure water to the cities of Europe was not an experiment; the sanitary campaign of Wood in Cuba was not an experiment; state medicine of today is not an experiment, and investments under its direction are not speculations.

### HEAR THE CONSTITUTION.

We, the people of the United States, in order to form a more perfect union, establish justice, insure domestic tranquility, provide for the common defense, promote the general welfare, and secure the blessings of liberty to ourselves and our posterity, do ordain and establish this constitution for the United States of America—to promote the general welfare.

The efficient sanitary organisation of our country, where the humblest citizen is a member of the royal family, will not be suitably inaugurated until there shall be in the cabinet at Washington a secretary of health. The medical profession of the United States would require but a few national campaigns in the name and interest of State Medicine to accomplish this most salutary, most desirable of sanitary achievements.

### PRACTICAL OBSERVATIONS.

The absolute justice of the state enlightened by the perfect reason of the state—that is law.

Reason is the life of law; nay, the common law itself is nothing else but reason.

Let us consider the possibility of having in our state a medical profession, united and organised for the purpose of State Medicine, and for such other purposes as experience and wisdom may deem advisable.

What are the essentials of such an organisation? They are three: (1) a common belief in principles so clear, so cogent, so logical, so practical that it will engage our minds, strengthen our convictions, stimulate our ambitions and warm our hearts until conviction may ripen into action; (2) an occasion calling for the efforts of such an organisation; (3) men to engage in the work who are urged on by a conviction of its necessity and eager for the labor it demands.

The art and science of State Medicine, the present needs of our people, our enormous death-rate from diseases known to be communicable and therefore preventable, the necessity of a higher standard for state license, furnish the occasion for the required opinions, knowledge, and convictions.

We are here. We are officials of the government. In the mind of the state we are one. We are working at the problems of state medicine, and in spite of our lack of unity we have made some progress toward their solution. We still believe that in union there is strength. The proposed unity, without requiring us to surrender any principle, prerogative or opinion, furnishes an opportunity of showing to the world a conspicuous instance of obedience to the law, of deference to the will of the sovereign people at the cost of some small sacrifice of custom, prejudice or pride.

It is significant that medical unity is tacitly recognised by ourselves as present in a greater degree than some would admit—from filing a certificate as to the cause of death all the way up to the highest function performed by a medical man, service upon a board of state examiners for state license.

We may well give a moment's consideration to the frame of mind on our part which is essential to a profitable consideration of this subject. Two things seem to be needful: (1) a thorough understanding of the temper of our legislators expressed in the medical laws of the state, together with an intimate knowledge of the law itself, and of the philosophy which underlies it and will be sure to influence any and all of its future developments and amendments. There is not time to treat the subject exhaustively and I can only advert to a single principle which seems to me the more important. The theory of our medical law has been, is, and will be, that to the people the medical profession is a unit. There may be, as there has been, varieties of belief, but there must be a common responsibility. The law never has undertaken and never will undertake to discriminate and choose between different medical beliefs and opinions regarding the mode of action of a given drug, or the method of treating a given disease. Upon such questions the law is silent, justly discerning that they are for the decision of medical men, singly, or in assemblies where varying minds and varying experience may hear and determine, and where if wrong conclusions are adopted, further observation, further experiment and fuller light, may make it the plain duty of today to reverse, as so many times has been done, the findings of yesterday.

Again, it seems to be essential that we should show a sympathetic loyal obedience to the law of the state both in its letter and its spirit. These words sympathetic, loyal obedience, are not lightly selected, but seriously chosen, for the obedience in question must be from the heart; it must be a sympathetic obedience, not the obedience of the officer who hates the regulations he is forced to observe, but that of the loyal Jesuit who obeys because he loves.

Finally, to this knowledge of the law, and to this condition in our hearts of sympathetic obedience thereto, must be added as a requisite to that state of medical organisation, whereby we alone may meet and discharge our full responsibility as the guardians of the public health, an abiding conviction of the need of medical unity. Remember that in stewards it is required that a man be found faithful.

Only to those of us fortunately possessed of these qualifications of knowledge, mind and heart, is this argument addressed; only to such does it seem possible that a wise conclusion may follow a judicious consideration. To others it will be like attempting to present the arguments of christianity to those who disbelieve in the existence and immortality of the soul.

Again, it may be stated as a general principle, that medical unity will never be made easier, brought nearer or made more efficient, by dwelling upon the limitations, or the imperfections in conduct or practice of our brethren. As long as we remain men such limitations and imperfections will abound. We ourselves may possibly at times show this evidence of a common humanity, and our brother's infirmity is but one more reason why he should have the advantage of our strengthening and elevating society.

Wisdom and experience teach that it is expedient for us to make much of our brother's good qualities, and that as to his faults forbear to judge, for we are sinners all.

We have seen that a successful organisation must have the continuous stimulus of work which calls for the doing, work which must be done, work which may be done only by the organisation in question.

Does such exist here and now? Surely it does. First of all, the best efforts of the organised medical profession of this state are needed to extend to its millions of people the protection, the preventive protection, of the demonstrated knowledge of hygiene.

It may not be our proud privilege to be pioneers and discoverers of new truths in any of the many departments of hygiene, but at least it is our duty to keep abreast of the column of progress and discovery, to be ready to receive the advances, to take advantage of the discoveries, to transmit to our people the priceless sickness preventing and life-saving hygienic truths which are being coined from day to day by the army of scientific pioneers,—remembering that we are just entering upon a new century, and that the highest round of successful accomplishment in one age is frequently only the point of departure for that which is to come.

The limits of your courteous forbearance and consideration for the proprieties of the occasion, do not permit an extended presentation of the manifold questions of hygiene that now press for solution. An entire address might well be devoted to the consideration of a single topic. It is enough to say that each is important, and that the whole constitutes a body of work, of transcendent moment, work in a realm in which the coin is not silver or gold, but that infinitely more precious material —human life.

Let me invite your attention to a group of communicable diseases which differ in destructive power and in the manner of communication, but are alike in their diffusion and alike in the manner in which their prevention is neglected,—gonorrhea, syphilis and consumption. From this one group, mentioned in a single breath, we suffer an annual mortality and stagger under an everlasting load of affliction and maiming almost incredible.

To attack this single group of diseases with success, demands knowledge, resources of legislation and command of capital in sovereign amounts. Here is an opportunity for every physician and philanthropist in the state to engage in work which can be accomplished only by and through the most thorough of organisations.

Intimately related to the foregoing in more than one way is the interesting and important sanitary problem of a better housing of the poor in our larger cities.

This subject is already a nightmare to those who study the causes operating to form the congested areas in our growing cities, and who realise what a menace to health and morals, to right living and to good citizenship such congested areas always are. Here, as in the previous problem, resources of the most varied character, ability of the highest order, zeal and perseverance second to none are required. Three thoughts should move us to prompt action: (1) the belief on the part of all students that prevention is easier and more economical than cure; (2) the conviction of the best informed that in many instances the growth of our cities has but begun; (3) the general consensus of feeling that the present century is to be noted for its success in the attack of hitherto resistant sociological problems, and that of such problems the better housing of the poor in cities is of preëminent importance.

For years those having authority have directed attention to the impure state of the waters in many of our streams, and the consequent prevalence of typhoid fever not alone, or chiefly, in large cities, but in many of the smaller towns. Attention has also been called over and over again to the important relation between the health of the community as well as to the productivity of the soil, and the protection from floods of a due proportion of forest lands. We know that in nature's great storehouse, where provision is made for the preservation of health and restoration from disease, no items equal in value pure water, pure air and sunshine. To keep our streams and other sources of water supply free from foulness and infection, to preserve our forests from useless destruction, to redeem denuded regions by reforestration, to drain our marshes where breeds the disease bearing anophiles, to transform the foul disease-spreading dampness of the sub-soil in our city streets into the benign, protective, ornamental foliage of the Carolina poplar, the elm or maple,—this is a work worthy of kings, a work that only kings may do, a work that the sovereign people of the Empire State may only accomplish when inspired and led by the united profession of medicine.

Without doubt, the most important event in our medical history of the last half century was the institution of state examination for license to practise the healing art. The immense significance of state license is in the fact that there we see the coöperation of the sovereign people and the united medical profession, the laying on of hands of pontif and profession, the blessing and the commission of the majesty of the law, the will of a free people, blended and united with the spirit of the art of healing, the most humane among all the arts and sciences; the recipient of the state license bears an honorable commission to a noble vocation.

Agreeably to the legitimate expectation, coincident with the establishment of state license to practise medicine an evolutionary revolution took place in the medical schools of the country. Before the time under consideration many of the graduates of our medical schools were prepared to enter upon an honorable career in medicine, but some of the graduates of such schools were indifferently prepared to get an easy living in what they supposed to be a lucrative trade. To prepare men for the medical degree of the college was one thing, to prepare them for the state examination quite a different one. For the former, two short terms and a few—less than a dozen—teachers would suffice; for the latter, four long terms and many dozens of teachers were required.

For the former, a medical faculty in every hamlet was the cry; for the latter, let us unite and found a great university is the word, and the joining of hands of the sovereign people and the united profession in state licenses is but a thing of yesterday; of a single decade. We may be well assured that no single responsibility is comparable with that of cherishing and developing and extending the principle of state examination and license; to it must continue to flow our best thoughts, our highest endeavors, for from it must come, if come it may, that high standard of personal and professional excellence and preparedness, which alone may enable us to cope with the ever increasingly intricate problems of modern medicine.

This commanding occasion gives me an opportunity to remind you, that there are in this state three examining boards where there should be but one; that the responsibility for this useless multiplication of official machinery rests primarily upon this society; that the people are aware of no good reason for this confusion; have but a scant and grudging patience with it, and that it is within our power, and in my judgment is our bounden duty to take the proper steps to make medical unity in state examination, unity in form as well as unity in spirit.

With a single board of examiners, representing the entire medical constituency of the state,—the entire lawful profession of the state, and therefore supported by the united medical profession,—we would be for the first time in position to expect and require of state examination a higher and still higher performance of its discriminating and selective functions. To some of the further practical demands upon our system of state license, demands of today, let us now for a few moments direct our attention.

I am informed that it has been from the first the wise policy of our state boards to raise the standard of examination from year to year slowly, but steadily; this course has without doubt been conservative and wise, and the large proportion of rejections indicate that the work of our teaching bodies is still far below the standard of the licensing body. However, it may be observed that the present standard of examination for license is not greatly different from that which obtained when the examination was inaugurated, whereas the provisions of our teaching faculties between then and now have changed greatly. Then terms of three months in two years, with lectures from a few teachers was the rule; now terms of six months, for four years, with instruction from scores and scores of teachers is the mode.

Is it not apparent, that the time has come, or will soon come, when it will be expedient to raise the standard for license in this Empire State so high that we may with truth declare of it, that there is none higher?

At present the severity of the test exacted by our examining boards of candidates for the state license is distinctly less than that for entrance to the medical service of our army, navy or marine hospital corps—is distinctly less than that of state examination or licensing examination in the chief countries of Europe. And yet the subjects of Edward or of William, of Italy or of France, neither deserve nor are able to reward better medical services than do the citizens of this state; it would be an easy and delightful task for a single state examining board supported by the united medical profession to raise the efficiency of that profession in this state so high that all might know and feel, that in illness, in accident, or in childbirth, every citizen, even the humblest dweller in the city tenement or in far away forest log house, would receive the care and attention of a medical man of unquestionable ability.

Again, it is to be noted that the medical service of our state is by no means small in number or insignificant in importance. That a special examination should be held to test the fitness of candidates for this service, is a question deserving serious consideration. Possibly the growth and multiplication of specialists has not yet reached its limit and the specialist in hygiene may yet appear.

Certainly it is an important part of the duty of state examination to provide for the highest possible efficiency in all departments of the medical service of the state. The need of this requires no further argument than the bare enumeration of the more familiar of these positions. Our health officers, state and local, are charged with enormous responsibility, in the direction of patronage, the disbursement of funds, the protection of health, the prevention of disease; they are not known to be especially fitted for their onerous duties. It is generally believed that their efficiency might be increased. Our commonwealth and municipalities annually expend enormous sums in the support of institutions for the treatment or maintenance of the blind, the deaf and the dumb, the orphan and destitute, the insane, and the criminal; the medical service of these institutions is of vast importance pecuniarily, professionally, sociologically, and these medical men and women have received no special preparation for their work. Who will say that such special preparation might not well be undertaken, and that special fitness might not be properly tested by special examination by the state examiners?

Again, it is a matter of common knowledge, that the work of our coroners, and postmortem examinations made for our coroners, do not always show that order of expert preparation desirable. Undoubtedly economy in the administration of the law, precision in the determination of medico-legal cases would result if the office of coroner was always filled by a medical man especially prepared for his work; and medical men at least would have more confidence in the reports of autopsies if they knew that men authorised for such work had made special preparation, and had given evidence of fitness, by passing with success a special examination by the board of state examiners.

Finally, we may not conclude these observations without adverting to the growing interest on the part of people and profession in the subject of school hygiene, and of medical inspection of our schools to the end that the health of the scholars and teachers may not be prejudiced by unhygienic conditions, and that the schools may not become the centers of distribution of the more common communicable diseases by reason of the attendance of pupils having the same. Here is work of singular importance, that possibly only awaits the appearance of the men and women properly prepared for it, whose fitness has been ascertained and certified by the proper authority.

It is confidently believed that the several subdivisions of medical practice before recited, constitute what might easily grow into a specialty of commanding importance, and of particular interest to the state and to preventive medicine, possibly sufficient to warrant the notice of our teachers of hygiene and of our state examiners. These practical observations are submitted for your consideration in the profound conviction, that the time is ripe for a sanitary organisation to engage in the work of forwarding the interests of the public health, to include the joined forces of the sovereign people, and the united medical profession of the Empire State.

### SCIENTIFIC.

The diagnostics and the prognostics of the maladies of nations, these are the largest questions pertaining to this world, that can be pondered by the mind of man.

That all the dead, the living, the unborn, are one moral person, one for suffering, one for action, one for responsibility.

There is that in American character which has never yet been found equal to its purpose. There is that in American enterprise which shrinks not, and faints not, and fails not in its labors.

Your attention has been invited to observations of a more or less political nature, and again to those of a complexion more distinctly practical; it remains to lay before you certain conclusions arising from a contemplation of our subject upon a background of the science of medicine.

The science of medicine! Who of all our number does not feel his heart quicken with pride at the very sound of the words?

Who of us in years past middle life does not recall the heart-sickening horrors of our early practice, when confronted with the signs of early pulmonary tuberculosis, realising its inevitably fatal termination; or of laryngeal diphtheria in a child under five years of age, knowing our helplessness, and the hopelessness of the case, and the agonising scenes of the slow suffocation to death from laryngeal stenosis; or the signs attending acute peritonitis, remembering from sad experiences, from reading, and from instruction, the enormous proportion of fatalities in the wellnigh hopeless malady. How thankful we are that having lived to see and endure so much of the uncertainty, the helplessness, the hopelessness of medicine of thirty years ago, it has been graciously vouchsafed to us also to live and to work in a period when science has put into our hands such vast powers of making early and accurate diagnoses, and modes of treatment, surgical and therapeutical, of such immense power and life-saving efficiency. Thanks! and thanksgiving to Almighty God, just and honorable pride in our noble profession, laud and praise and everlasting honor to the men who have made, who are making, and who are yet to make the science of medicine; these sentiments are becoming and appropriate to the subject and the occasion.

Your attention was directed to the remarkable development which has recently taken place in our medical schools, a development which has already worked a substantial transformation in our teaching faculties, their equipment for work, and their mode of teaching. Without question this development is normal, is growth, is the product of natural forces, is to be permanent, and has but fairly gotten under way. In connection with this, three thoughts deserve our earnest attention.

Medicine, as practised at the bedside, or in the consulting room, has ever had a blending of science and art; the mode of teaching medicine from the days of Hippocrates to the last decade of the last century has been that of men who saw and hoped great things of the art and knew but little of the science. Our profession, nevertheless, has been free in spirit and has profited immensely by the contemporaneous advances in the allied sciences of chemistry, physics and biology, has contributed to the glorious galaxy of heavenly bodies a star of the first magnitude—the science of bacteriology, and by the light of this new luminary is rapidly advancing in the hitherto most discouraging of all her fields, and is day by day adding triumph to victory, triumph in diagnosis, and victory in treatment. With the slow but surely increasing illumination of this department of practice, by the rising sun of scientific therapia, medicine may declare herself to be scientific in all her ways. The long and honorable era of the empiric teaching of the art of medicine has closed; the twentieth century finds the era of the demonstrative teaching of the science of medicine auspiciously inaugurated.

The importance of medical unity and of relations of confidence and sympathy with the people becomes manifest when we contemplate the cost for equipment and for maintenance, of numerous teachers, the vast libraries, the museums of specimens anatomical, physiological and pathological, the laboratories, chemical, biological, pathological and bacteriological, together with the facilities of hospitals and dispensaries, some of the prime necessities for the teaching of medicine of today. From Hippocrates to Koch is a long stretch in time and in method, but it is not greater than the difference in method and equipment between the medical college of the nineteenth century and the medical department of the university of the twentieth century.

Heretofore, we have directed your attention, not without conscious pride in the opportunity, to the fact that the spirit of medicine is free, that she wisely borrows, to profit withal from the discoveries and advances of the contemporaneous sciences. During the final decades of the last century the science of psychology has come into prominence, and has made invaluable contributions to the ancient and honorable art of pedagogics.

In part as a result of this, and in part from independent observations the theories and the modes of teaching of today differ widely from those of yesterday—methods have changed greatly and will yet change. My knowledge of the methods of teaching current in our various medical schools is not sufficient to enable me to inform you how far medical teaching has benefited by the current advance in pedagogics, but it is my great privilege to be permitted to congratulate you upon our fortunate situation. The University of the State of New York, the inspiration and head of our magnificent educational system, licenses such of the graduates of our medical schools as successfully pass the state examination. As directed by law, accurate record is kept of the standing of those examined, with records of those who fail to reach the standard requirement.

These records from year to year throw a beam of white light upon the methods, efficiency and success of our different teaching bodies, emphasising those whose pedagogical methods are deserving and winning success, and giving admonition and warning to others; that the struggle for existence is not confined to plants and animals, that in medical colleges only the fittest are likely to survive, that if our state standards of this decade are sometimes found to be inconvenient, those of the next decade may prove to be prohibitive. The question of medical teaching which shall be in accordance with the best medical traditions and principles, and also along the best pedagogical lines, is closely related to the question of large foundations, of endowments of chairs and scholarships, and this in turn is vitally dependent upon the sympathy and good-will of those who are rich in this world's goods.

Hitherto, mention has been made of the tendency of the times to develop for purposes economical, commercial or financial organisations and combinations, involving capital, employment, and product in quantities and proportions well-nigh fabulous, certainly beyond the wildest dreams of the men of business of half a century ago. This condition is not accidental, neither is it as some of the more timid assert, a sign of degeneracy of the body politic; but it is the legitimate result of free institutions under auspicious environment operating to produce and to accumulate the kindly fruits of the earth for the betterment and the enjoyment of the people, in such surprising profusion, in such enormous quantities as to verily indicate that this is the very promised land—that when our forefathers and our brothers shed their blood that there might be liberty and union on this continent, they did in fact and in confident faith seek the Kingdom, believing in the promise—and all these things shall be added unto you.

Of this we may be assured that as history will describe the last century as the age of preparation, of accumulation, in like manner the present century will be known as the age of distribution of performance, of accomplishment. Indications are not wanting that the American stage is being set for the performance of an honorable part in the drama of life. Certainly wealth is accumulating here as nowhere else in the world, and emigration is pouring into our country a vitalising, energising, progress-making stream of human life, all with upraised faces, of greater significance, of more hopeful potentality than the combined products of our mines, our mills and our farms. Again it is to be observed, that the wealth of America is a consecrated wealth, consecrated in the highest and most intelligent degree to the beneficent purposes of constitutional government. The benefactions of our people in the last decade for charitable educational, scientific and allied purposes, is an almost incredible sum, and is a plain indication that the enormous wealth of the country is in the hands of men of high and noble principles.

The problems of the new century are before us. Which of those problems compares in interest, in significance, in importance to the welfare of the people, the maintenance of free government, to the progress of our race, with the scientific problems of state medicine?

How may we better prepare to meet those problems, than by striving for medical unity; hoping thereby to gain somewhat in numbers and influence and to improve distinctly in form—that our purposes may be the better understood by the people, that our institutions may be prepared to train men to the full stature of the science and art of medicine, that our legislature may enact suitable laws for the due protection of the public health, and furthermore that the sympathy and interdependence between the profession and the people may become so intimate that our sanitary codes and ordinances shall be everywhere intelligently obeyed.

Let us hear the conclusion of the matter. *E pluribus unum! Excelsior*! The spirit of America. The thought of the Empire State. The Zeitgeist of the twentieth century. The science of medicine. Never before in the world have these mighty thoughts carried such possibilities of duty, of conviction, of action, as now. *E pluribus unum!* Our theme is the all sufficiency of the law, concerning which it has been written, Of law there can be no less acknowledged than that her seat is the bosom of God, her voice the harmony of the world; all things in heaven and earth do her homage, the very least as feeling her care, and the greatest as not exempted from her power. *E pluribus unum!* Thus, for more than a century. We are many, we may be one. We are many, with all the indirectness, the confusion, the inefficiency, that ever attends disunion and disorganisation. Let us become one, one for the defense of the public health, one for the uplifting of every member of our profession, one for the promotion of the most humane, the most inspiring the most beneficent of all material vocations, the science and art of medicine. *Excelsior!* When a regiment advances a company must take the lead. You hold in your hand a glass containing a solution of a given salt of a certain concentration. Your desire is that the salt shall evolve from the chaos of solution to the accurately, efficiently, organised form of crystalisation. You drop into the glass a single crystal of the given chemical and in due time chaos gives way to order, solution yields to crystalisation, disunion and disorder give place to union. The medical profession of America is without unity, or such organisation as becomes the free profession of a free and united people. What could be more appropriate, more inspiring, more eminently distinguishing, than the example of the Empire State, the state of the metropolis of all the Americas, the state of Alexander Hamilton, of Chancellor Kent, of John Jay, the state of enormous wealth, the state of marvelous achievements, of vast and bewildering possibilities, giving to the profession of America, an example of medical unity under the law !

433 Franklin Street.

